# Investigation of the causal relationship between inflammatory bowel disease and type 2 diabetes mellitus: a Mendelian randomization study

**DOI:** 10.3389/fgene.2024.1325401

**Published:** 2024-02-16

**Authors:** Ling-tong Tang, Lei Feng, Hui-ying Cao, Rui Shi, Bei-bei Luo, Yan-bi Zhang, Yan-mei Liu, Jian Zhang, Shuang-yue Li

**Affiliations:** ^1^ Department of Clinical Laboratory, Yan’an Hospital Affiliated to Kunming Medical University, Kunming, Yunnan, China; ^2^ Department of Clinical Laboratory, Sixth Affiliated Hospital of Kunming Medical University, Kunming, Yunnan, China

**Keywords:** Mendelian randomization, type 2 diabetes mellitus, inflammatory bowel disease, irritable bowel syndrome, causal relationship

## Abstract

**Background:** Type 2 diabetes mellitus (T2DM) and inflammatory bowel disease (IBD) have been associated, according to various epidemiological research. This study uses Mendelian randomization (MR) to investigate the causal link between T2DM and IBD.

**Methods:** To investigate the causal relationship between IBD and T2DM risk using European population data from the genome-wide association study (GWAS) summary datasets, we constructed a two-sample MR study to evaluate the genetically predicted impacts of liability towards IBD outcomes on T2DM risk. As instrumental variables (IVs), we chose 26 single nucleotide polymorphisms (SNPs) associated with IBD exposure data. The European T2DM GWAS data was obtained from the IEU OpenGWAS Project database, which contains 298,957 cases as the outcome data. The causal relationship between T2DM and IBD using a reverse MR analysis was also performed.

**Results:** The two-sample MR analysis, with the Bonferroni adjustment for multiple testing, revealed that T2DM risk in Europeans is unaffected by their IBD liability (odds ratio (OR): 0.950–1.066, 95% confidence interval (CI): 0.885–1.019, *p* = 0.152–0.926). The effects of liability to T2DM on IBD were not supported by the reverse MR analysis either (OR: 0.739–1.131, 95% confidence interval (CI): 0.651–1.100, *p* = 0.058–0.832). MR analysis of IBS on T2DM also have no significant causal relationship (OR: 0.003–1.007, 95% confidence interval (CI): 1.013–5.791, *p* = 0.069–0.790). FUMA precisely mapped 22 protein-coding genes utilizing significant SNPs of T2DM acquired from GWAS.

**Conclusion:** The MR study showed that the existing evidence did not support the significant causal effect of IBD on T2DM, nor did it support the causal impact of T2DM on IBD.

## Introduction

Type 2 diabetes mellitus (T2DM) is a chronic metabolic disorder defined by pancreatic β-cells failure and insulin resistance in peripheral tissues. This results in impaired glucose metabolism and chronic low-grade inflammation (Zhou et al., 2022). This chronic disease is one of the leading causes of death and disability in the world, which is caused by genetic and environmental factors, such as genetic predisposition, unhealthy diet, adiposity, smoking, ambient air pollution, physical inactivity, and pre existing underlying diseases are important reasons for its continuous increase in incidence (Almigbal et al., 2023; Arsh et al., 2023; Taborda Restrepo et al., 2023). According to a report, the global prevalence of diabetes among adults exceeded 460 million in 2019, with projections indicating a substantial increase to over 700 million by 2045 (Sun et al., 2022). Given its association with detrimental microvascular and macrovascular consequences, T2DM inflicts physical and psychological distress on patients and imposes a significant financial burden on the healthcare system (Kang et al., 2022; Alemayehu et al., 2023; Hands et al., 2023). Inflammatory bowel disease (IBD), encompassing ulcerative colitis and Crohn’s disease, manifests in around 1% of the population and is commonly distinguished by persistent diarrhea (with or without bleeding), stomach discomfort, and loss of body mass (Bruner et al., 2023). It is more common between the ages of 20 and 40, but can start at any age, resulting in significant differences in disease course and complications among different individuals, and the immune systems involved are also more complex, including the innate and adaptive immune systems (Caioni et al., 2021; Saez et al., 2023; Tseng, 2023). Numerous proinflammatory immune mediators, such as interleukin 17, interleukin 23, interferon gamma, and tumor necrosis factor alpha overexpression in it (Flynn and Eisenstein, 2019; Noviello et al., 2021; Parigi et al., 2022). It is linked to increasing damage to the intestine and extra-intestinal symptoms, resulting in compromised gastrointestinal function, reduced quality of life, and heightened therapeutic challenges (Dai et al., 2023). Due to the intricate and partially unknown etiological origins and development of ulcerative colitis and Crohn’s disease, effectively managing these conditions can present difficulties, both in terms of clinical perspectives and resource allocation (Da Rio et al., 2023). In the literature, IBD has been documented to exhibit associations with several medical conditions, such as colorectal cancer (Gordon et al., 2023), Graves’ disease (Xian et al., 2023), and metabolic disorder (Verdugo-Meza et al., 2020).

Previous studies have observed that IBD is a chronic and idiopathic inflammatory condition affecting the gastrointestinal system. It has also been linked to T2DM, according to various studies (Zhao et al., 2021; Allin et al., 2022; Tseng, 2022). The primary anatomical location of IBD is the large intestine, which has the highest concentration of bacterial cells. Research conducted on the gut microbiota in individuals with IBD around the globe has revealed that dysbiosis, characterized by alterations in the composition of intestinal bacteria, is associated with either an increase or reduction in certain bacterial species inside the gut of IBD patients (Al Bander et al., 2020). Alterations in microbial homeostasis in the intestine have profound implications for local and systemic immunity, hence significantly developing extra-intestinal systemic disorders such as obesity, diabetes, and atherosclerosis (Gill et al., 2022). Some epidemiological studies showed the potential relationship between T2DM and IBD. Villumsen et al. (2022)found that inflammatory bowel disease would increase the risk of type 2 diabetes. Abrahami et al. (2018) pointed out that the use of Dipeptidylpeptidase-4 inhibitors (DPP4i) in T2DM was related to the increased risk of IBD, but (Kim et al., 2015)reached the opposite conclusion that starting DPP4i in T2 diabetes could reduce the risk of IBD. But, the causality of these correlations has yet to be established. The presence of unmeasured confounding and reverse causation in this epidemiological research introduces bias, which poses challenges to establishing causal inference. However, investigating a potential causative relationship between IBD and T2DM might provide valuable insights into specific biological mechanisms and contribute to developing effective preventive measures. Mendelian randomization (MR) is an epidemiological methodology that addresses many biases commonly seen in observational research, including reverse causality and confounding (Gagnon et al., 2023). It also utilizes genetic variants strongly associated with the exposure that satisfies certain assumptions as IVs to investigate the causal relationship with an outcome. (Yin et al., 2022). Given that these variations are assigned randomly during conception, this might mitigate bias resulting from environmental confounders, provided that MR is carried out appropriately. Therefore, the MR design may be comparable to a randomized controlled trial. In this work, we use a Mendelian randomization design to investigate the causal relationship between a specific exposure (in this case, IBD) and an outcome (in this case, T2DM) by utilizing an IVs approach, as described in the aforementioned technique (Tseng, 2021). We utilized bidirectional MR to investigate the presence of a causal relationship between IBD and T2DM. This was accomplished using summary data from the most extensive genome-wide association studies (GWAS) conducted on European populations for the aforementioned diseases.

## Materials and methods

### Overall study design

**FIGURE 1:**
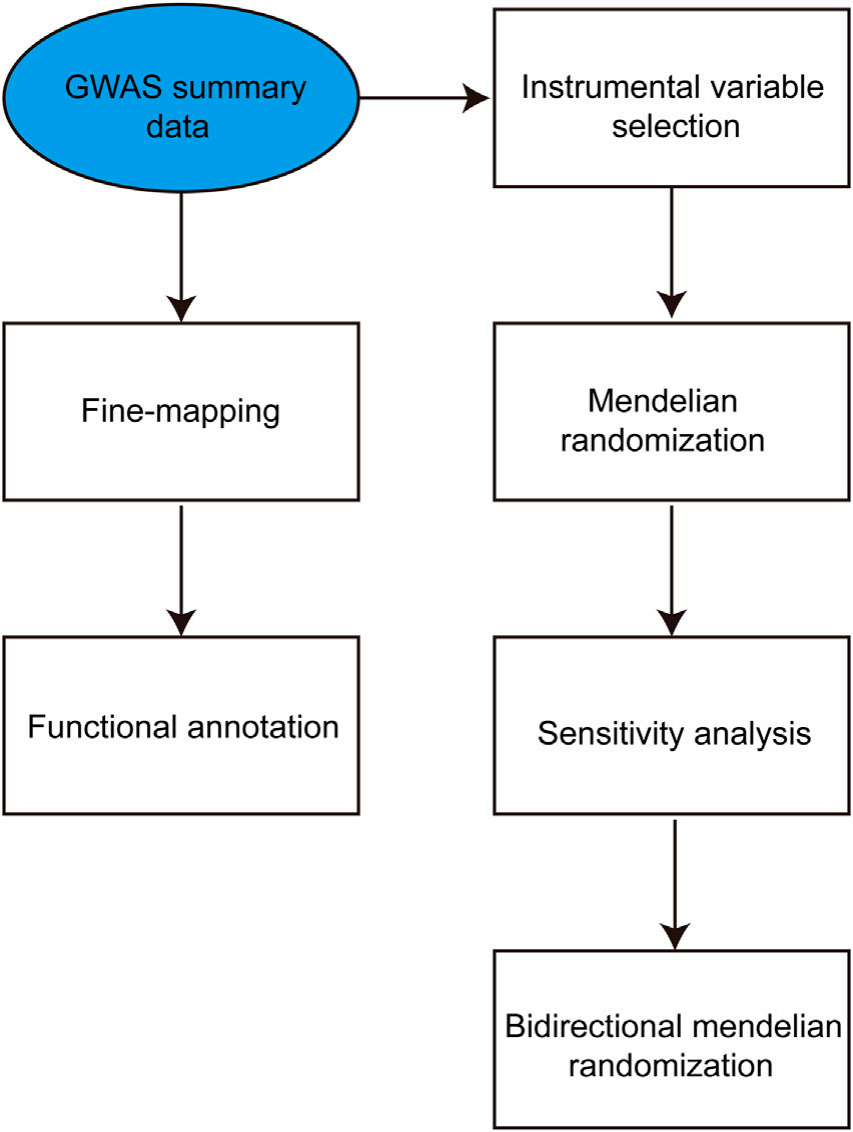
The study design and Mendelian randomization methods used in the analysis.

Initially, the summary statistics of genome-wide association studies on IBD and T2DM were obtained from the GWAS Catalog. Following this, an extensive bidirectional two-sample MR analysis was conducted to examine the causal associations between susceptibility to IBD and T2DM. In the first phase of the analysis, the exposure variable was IBD, whereas the outcome variable was T2DM. In the second step, the exposure variable was T2DM, and the outcome variable was IBD. Subsequently, we employed IBD as the exposure while IBD and T2DM were considered the outcomes for conducting a two-sample MR analysis. The study design is depicted in Figure 1.

### Data retrieval

Based on the search results of datasets from GWAS on IBD (GCST003043), it had summary statistics for a total of 126,096 single nucleotide polymorphism (SNP) (Liu et al., 2015). The present GWAS meta-analysis investigation revealed datasets consisting of 34,652 individuals of European ancestry. A replication dataset was included, comprising 6,543 individuals of East Asian descent, 890 individuals of Greater Middle Eastern origin (including Middle Eastern, North African, or Persian populations), 51,988 individuals of European descent, and 2,413 individuals of South Asian ancestry. The GWAS dataset for T2DM (GCST007517) had summary statistics for a total of 131,218 SNP (Mahajan et al., 2018). The present GWAS meta-analysis was conducted on a sample of 298,957 people of European descent. The original publications provide comprehensive information on recruiting techniques and diagnostic criteria. The GWAS data utilized to analyze IBS (Dönertaş et al., 2021). This dataset encompassed a total of 9,689,034 SNP derived from 484,598 samples. The data was collected from the GWAS Catalog, specifically identified by the data number GCST90038626. SNPs location information using the human reference genome GRCH38 version. Information on recruitment procedures and diagnostic criteria is detailed in the original publications. Case-control association tests for IBD, IBS and T2DM were performed in each group using a linear mixed model as implemented in MMM (Pirinen et al., 2013). Moreover, as the GWAS samples were independent, no overlap is observed between the groups in GWAS populations.

### Instrumental variable selection

The analysis focused on the genetic data from GWAS on IBD as the exposure factor. Initially, the selection of suitable SNPs as IVs was conducted by using the Bonferroni-corrected *p*-value obtained from multiple testing, as well as a minimum allele frequency (MAF) threshold (*p* < 5e-8, MAF >0.01) (Yuan et al., 2023a). Subsequently, the genetic linkage coefficient of commonly occurring SNPs in the European population reference panel (1000G phase III EUR), as made available through the R package IEU GWAS, was employed to exclude SNPs that exhibited strong linkage. This identification process was carried out using a clumping window of 10,000 kb and an *r* ^2^ cutoff value of 0.001, as described in a prior study (Xiang et al., 2021). Ultimately, SNPs that were present in both the exposure and outcome GWAS summary statistics and had available data were selected as the final instrumental variables (Table 1). The GWAS summary statistical data for IVs is presented in Supplementary Table S1. The reverse MR was assessed following the same procedure.

**TABLE 1:** Information on instrumental variables (IVs) subjected to inflammatory bowel disease (IBD).

Rs number	Chromosome	Location	Other allele	Effect allele	EAF	*p*-value
**rs10761659**	10	62,685,804	A	G	0.54	4.97E-53
**rs11230563**	11	61,008,737	C	T	0.35	1.71E-14
**rs1535**	11	61,830,500	A	G	0.33	2.78E-09
**rs3184504**	12	111,446,804	T	C	0.51	1.29E-09
**rs941823**	13	40,439,840	T	C	0.75	6.19E-13
**rs17293632**	15	67,150,258	C	T	0.24	2.71E-20
**rs744166**	17	42,362,183	A	G	0.42	1.14E-22
**rs1292053**	17	59,886,176	A	G	0.44	9.89E-13
**rs12720356**	19	10,359,299	A	C	0.09	4.13E-16
**rs3806308**	1	19,816,373	C	T	0.38	1.08E-21
**rs34856868**	1	92,088,726	G	A	0.03	9.80E-09
**rs10800309**	1	161,502,368	A	G	0.66	6.16E-37
**rs3024493**	1	206,770,623	C	A	0.16	1.65E-50
**rs6074022**	20	46,111,557	C	T	0.75	8.32E-11
**rs780094**	2	27,518,370	T	C	0.61	3.88E-15
**rs7608910**	2	60,977,721	A	G	0.39	2.60E-36
**rs1990760**	2	162,267,541	C	T	0.61	3.56E-10
**rs406113**	6	28,515,705	A	C	0.32	4.06E-08
**rs4151651**	6	31,947,837	G	A	0.03	1.13E-51
**rs3807039**	6	32,110,596	A	C	0.11	9.67E-19
**rs3806157**	6	32,406,024	T	G	0.35	3.28E-13
**rs1847472**	6	90,263,440	C	A	0.34	6.63E-10
**rs1182188**	7	2,830,351	T	C	0.30	1.08E-09
**rs2108225**	7	107,812,658	G	A	0.44	1.27E-11
**rs6651252**	8	128,554,935	T	C	0.13	9.08E-10
**rs10758669**	9	4,981,602	C	A	0.65	4.70E-48

### Mendelian randomization (MR) analyses

The TwoSample MR R package was utilized for the bidirectional two-sample MR (Li et al., 2022). We used IBD as the exposure and T2DM as the outcome to build two-sample MR model. Then, to integrate the influence of distinct IVs, the inverse variance weighted (IVW) method was employed (Huang et al., 2022). The IVW technique was mainly used for fundamental causal estimations, which would yield the most accurate findings if all chosen SNPs were acceptable IVs (Xu et al., 2022). For each SNP, the Wald ratio was determined, and the individual effect of each SNP was meta-analyzed using IVW to get the final beta estimate, which was transformed into an OR (Alipour et al., 2022). To evaluate the third MR assumption, the MR Egger analysis was applied to identify any violations of IV assumptions owing to directional horizontal pleiotropy (Luo et al., 2022). We use the mr_pleiotropy_test function of R package TwoSampleMR (v0.5.7, https://rdrr.io/github/MRCIEU/TwoSampleMR/) to test the pleiotropy of SNPs.Cochran’s Q test assessed heterogeneity in the IVW and MR Egger procedures. We use R package TwoSampleMR’s MR_ The heterogeneity function to calculate the Cochran’s Q statistic, the following formula: $Q=\sum_{j}w_{j}{\left({\hat{\beta }}_{j}-\hat{\beta }\right)}^{2}({\hat{\beta }}_{j}$ is the estimated coefficient value obtained from the *j*th IV, $w_{j}$ is the corresponding weight, and $\hat{\beta }$ is the pooled estimate value obtained by combining IVW or MR Egger), where two Q statistics refer to IVW and MR Egger with *p*-value <0.05 were deemed to be reliable. The Weighted Median regression method, calculates a weighted median of the Wald ratio estimates and is robust to horizontal pleiotropic bias, when the majority valid assumption holds. (Bowden et al., 2016). It has been verified that the Weighted Median approach outperforms the MR-Egger regression regarding lowering the type I error and higher causal estimate power (Ding et al., 2023). Finally, a leave-one-out (LOO) analysis was undertaken to determine whether any particular SNP is disproportionately responsible for the outcome of any MR study (Gao et al., 2023). Applying Bonferroni correction for multiple testing, a *p*-value below 2.1E−03 was considered as signifcant.

### Post-GWAS analysis of type 2 diabetes-associated SNPs

We expect to observe associations with genes involved in T2DM, so we used FUMA to perform a functional mapping of genetic associations to loci of the T2DM GWAS. Functional Mapping and Annotation (FUMA) is a comprehensive approach that combines positional mapping (Dai et al., 2022). The current study used FUMA, Expression quantitative trait locus (eQTL) mapping, and chromatin interaction mapping approaches, to perform precise mapping of SNPs identified in GWAS for T2DM. SNPs that exhibit a *p*-value less than 5e-8 are commonly referred to as tag SNPs. These tag SNPs are then included in the FUMA system for fine mapping (Cao et al., 2022).

Furthermore, the default parameters are utilized for position mapping, eQTL mapping, and 3D Chromatin Interaction mapping. The present study employed genes derived from fine mapping using MAGMA software for enrichment analysis by enriching them in various gene function sets and tissues, categorized based on GTEx’s 30 general tissues (Xu et al., 2023). Enrichment analysis (Gene Set Enrichment Analysis, GSEA) determines if a group of genes appears more frequently in a specific functional pathway than random chance (Yang et al., 2023). Using the precise test method of hypergeometric distribution, taking the enrichment analysis of differential expression as an example, the *p*-value calculation formula is: $p=1-\sum_{j=0}^{x-1}\frac{\left(\begin{array}{c} M\\ j \end{array}\right)\left(\begin{array}{c} N-M\\ n-j \end{array}\right)}{\left(\begin{array}{c} N\\ n \end{array}\right)}$ .Among them, N represents the total number of genes, n represents the number of differentially expressed genes, M represents the total number of genes in the gene set, and j represents the number of differentially expressed genes in the gene set. The tissue-specific expression of genes acquired through fine mapping was examined using FUMA, utilizing the 30 general tissues provided by GTEx v8 (Yuan et al., 2023b). Heatmaps depicting the variations in gene expression unique to distinct tissues were produced and analyzed to identify differentially generated (Li et al., 2023). In a manner akin to the preceding enrichment analysis, additional functional datasets such as Gene Ontology (GO) and Kyoto Encyclopedia of Genes and Genomes (KEGG) were incorporated (Zhang et al., 2023). Integrating the GWAS Catalog into FUMA enhances the enrichment of identified genes within several biological functional modules and pathways. The objective is to ascertain the presence of functional modules within the gene set linked to T2DM and their potential correlations with other diseases or phenotypes.

## Results

**FIGURE 2:**
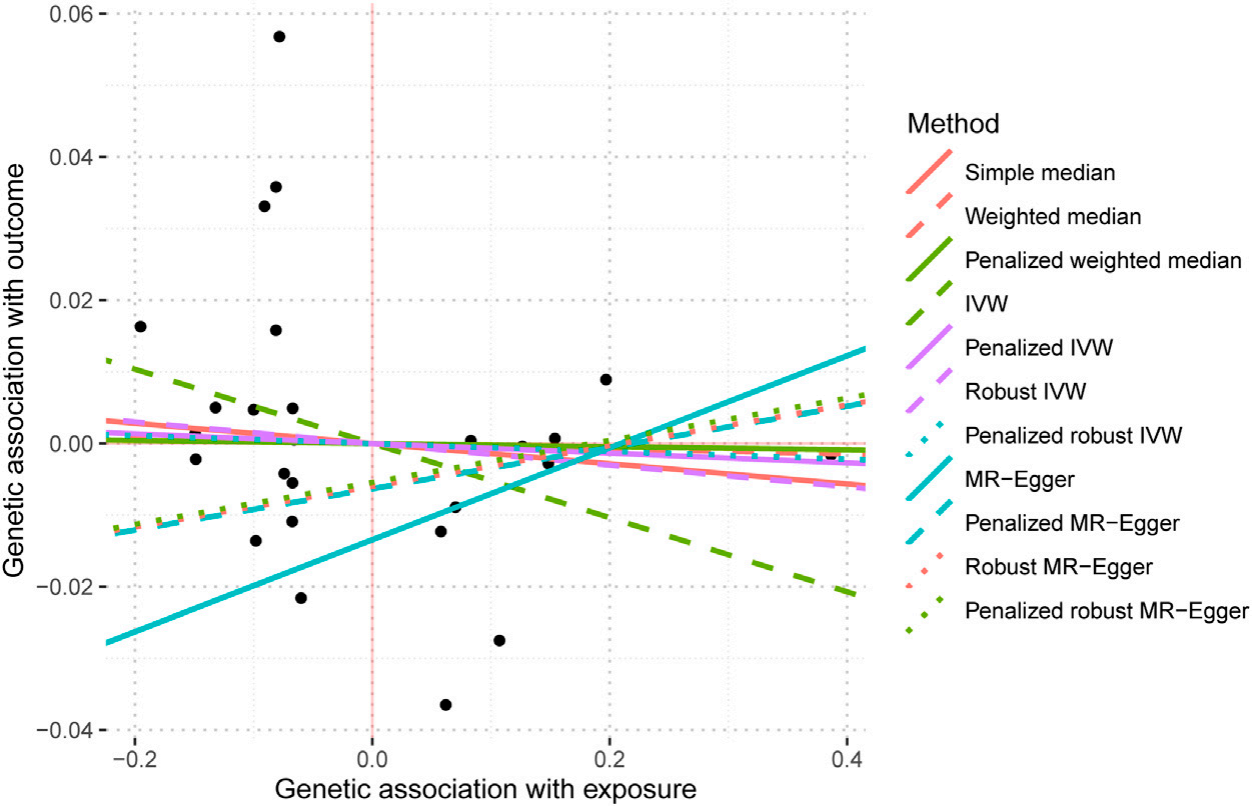
Mendelian randomization scatter plot diagram of two samples of the causal effect of IBD on T2DM. The *x*-axis represents the genetic association with IBD risk; the *y*-axis represents the genetic association with the risk of T2DM. Each line represents a diferent MR method. IBD, inflammatory bowel disease; T2DM, Type 2 diabetes mellitus; MR, Mendelian randomization. No signifcant associations were detected.

**FIGURE 3:**
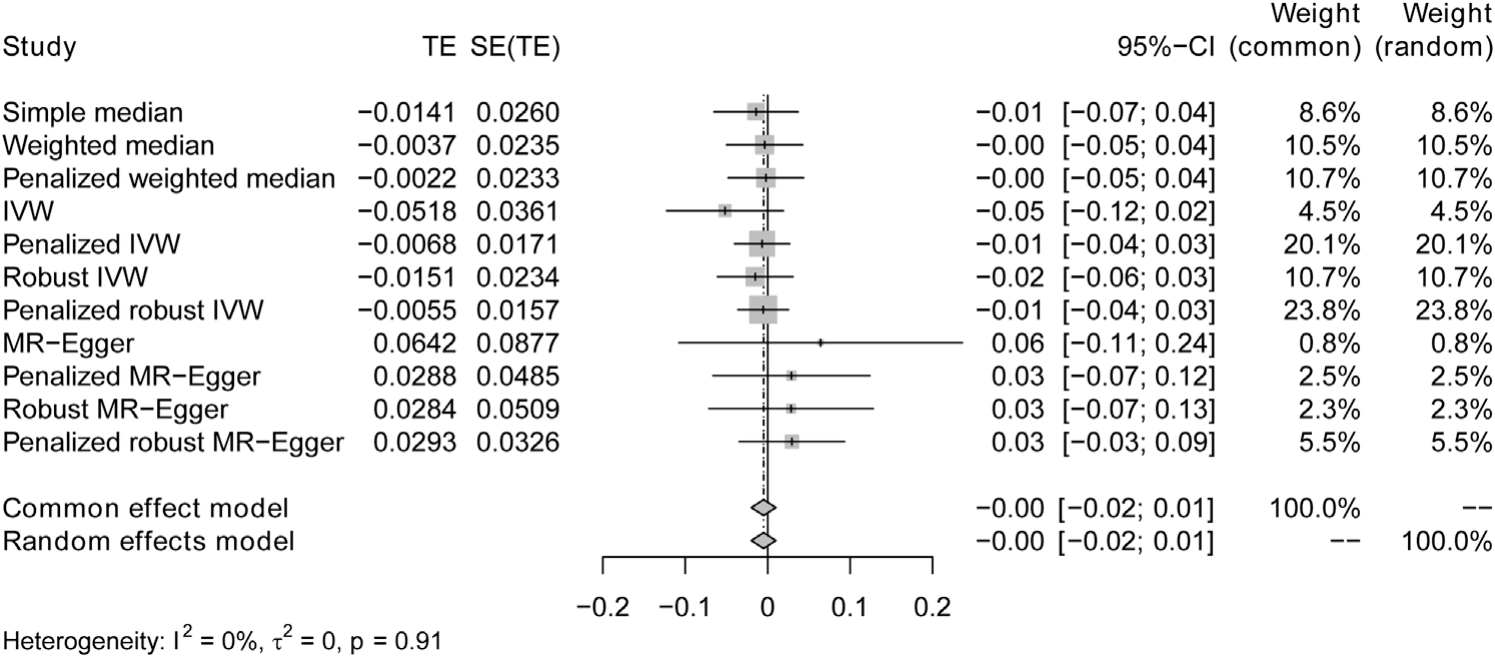
Forest plots of causal effects for IBD on T2DM. Summary of the Mendelian randomization (MR) result derived from the inverse-variance weight, MR Egger, and weighted median method, using the fixed effect common effect model.

The IVW analysis yielded findings indicating that the susceptibility to IBD does not have an impact on the risk of T2DM. These results align with the outcomes obtained by other MR techniques, such as MR Egger and weighted median, as presented in Table 2 and Figure 2. Table 2 displays the outcomes of the MR analyses that examined the causal association between IBD and T2DM. Correspondingly, Figure 2 presents scatter plots illustrating these findings. Based on the findings shown in Table 2 and Figure 2, it is evident that none of the approaches yielded statistically significant causal estimates when employing a significance level of 0.05. This finding can also be observed through implementing Forest Plots (Figure 3). Furthermore, the positive and negative values produced by different methods exhibited variability. In addition, the MR-Egger, the Penalized MR-Egger, the Robust MR-Egger, and the Penalized robust MR-Egger methods showed consistent positive results. Conversely, the Simple median, the Weighted median, the Penalized weighted median, the IVW, the Penalized IVW, the Robust IVW, and the Penalized robust IVW methods showed consistent negative results. The results indicate that there may be pleiotropy or heterogeneity among the Instrumental Variable used for MR Egger and IVW analysis. All in all, Mendelian randomization of two samples shows that MR analysis is insufficient to support the causal effect of IBD on T2DM.

**TABLE 2:** Estimation of causal effect of IBD on T2DM with different MR methods.

Method	Estimate	Std error	OR	95% CI		*p*-value
Simple median	−0.014	0.026	0.986	0.937	1.038	0.587
Weighted median	−0.004	0.024	0.996	0.951	1.043	0.876
Penalized weighted median	−0.002	0.023	0.998	0.953	1.045	0.926
IVW	−0.052	0.036	0.950	0.885	1.019	0.152
Penalized IVW	−0.007	0.017	0.993	0.961	1.027	0.691
Robust IVW	−0.015	0.023	0.985	0.941	1.031	0.518
Penalized robust IVW	−0.005	0.016	0.995	0.964	1.026	0.727
MR-Egger	0.064	0.088	1.066	0.898	1.266	0.464
Penalized MR-Egger	0.029	0.049	1.029	0.936	1.132	0.553
Robust MR-Egger	0.028	0.051	1.029	0.931	1.137	0.576
Penalized robust MR-Egger	0.029	0.033	1.030	0.966	1.098	0.368

* The ORs, express effects of liability to IBD, on T2DM, risk. OR, odds ratio; CI, confidence interval; MR, mendelian randomization.

**FIGURE 4:**
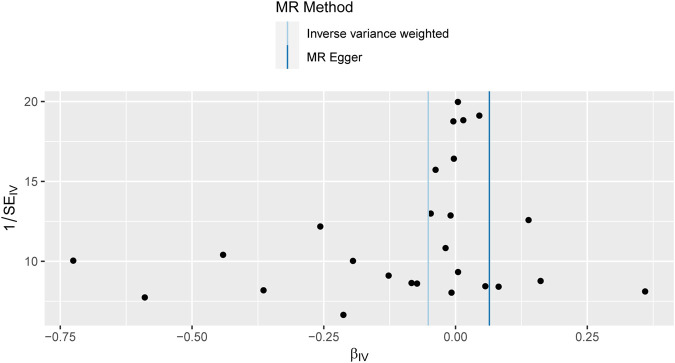
Funnel plot of Mendelian randomization results. Funnel plots for overall heterogeneity of MR estimates for the effect of IBD on T2DM. IVW, inverse‐variance weighted.

Sensitivity studies were conducted to identify the potential existence of horizontal pleiotropy. To validate the reliability of the aforementioned study findings, a sensitivity analysis was performed following the methodology outlined in the methods section. Initially, a pleiotropy test was performed, yielding a test statistic of *p* = 0.161,304, which is above the conventional significance threshold of 0.05. This outcome suggests no substantial pleiotropy, hence validating the choice of IV. Additionally, heterogeneity tests were performed on the IVW and MR Egger techniques, and the outcomes are presented in Table 3. Based on the outcomes of the heterogeneity test presented in Table 3, it was found that there was a notable presence of heterogeneity among the chosen instrumental factors (*p* < 0.05). Confirming this finding can also be observed through the funnel plot (Figure 4). These visual representations demonstrate that the estimated values derived from each SNP exhibit an asymmetrical distribution on both ends of the combined estimate. Additionally, there is a notable and statistically significant rightward deviation in the merged estimate.

**TABLE 3:** Heterogeneity test result on the IVW and MR Egger methods.

Method	Q	df	*p*-value
MR Egger	116.8911959	24	3.46E-14
Inverse variance weighted	127.0651,513	25	1.23E-15

**FIGURE 5:**
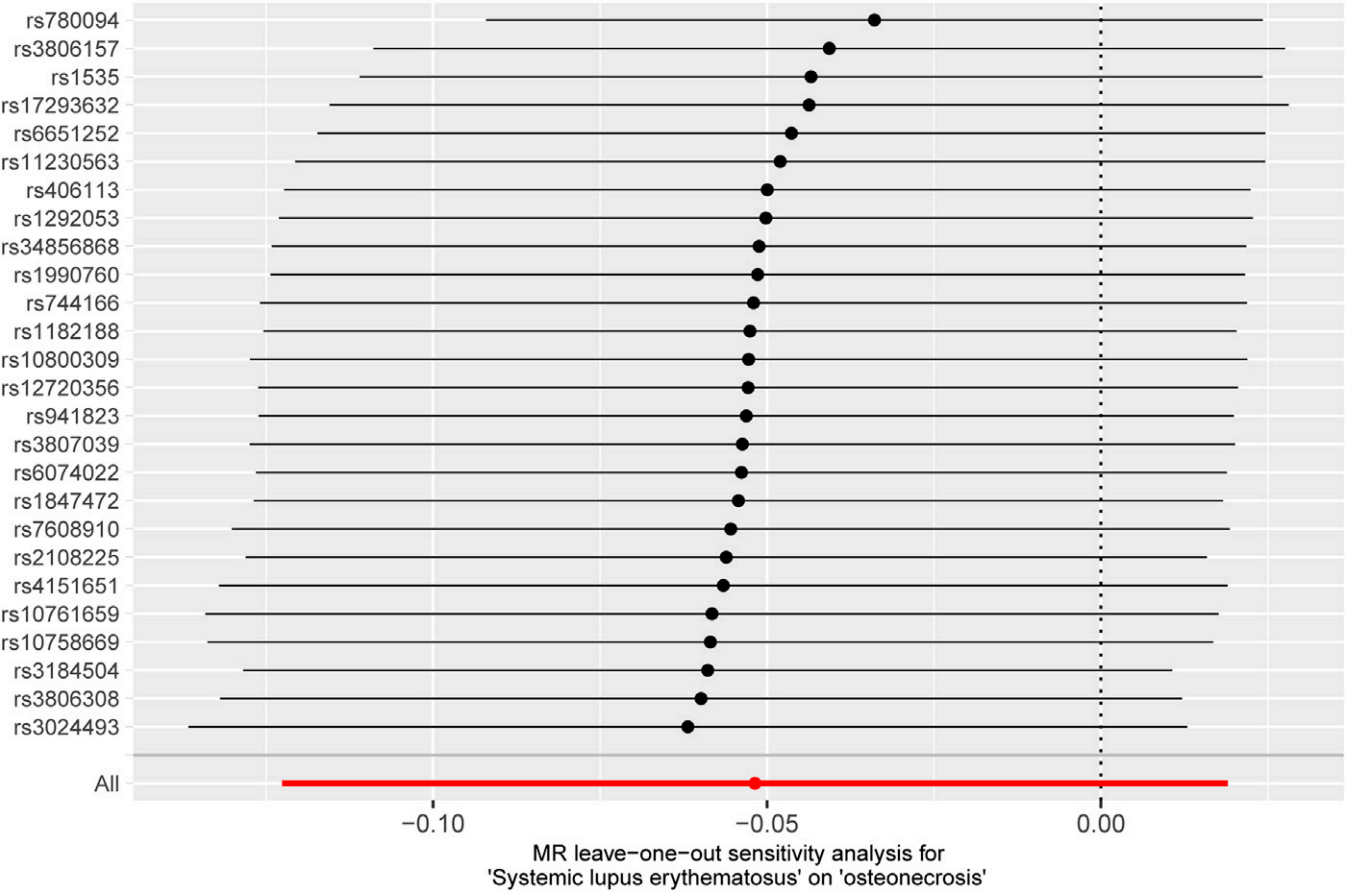
MR leave-one-out sensitivity analysis of T2DM to investigate the possibility of causal association driven by a particular SNP. Each black point represents an inverse variance weighted method for estimating the causal effect of IBD on T2DM, excluding that particular instrumental variable from the analysis. Redpoint represents the estimate using all instrumental variables. Horizontal lines denote 95% confidence intervals. OR, odds ratio; SD, standard deviation.

Finally, a sensitivity analysis was performed on the retention method. After iteratively removing individual SNP one by one, the estimated forest plot was obtained via IVW (Figure 5). As depicted in Figure 5, the estimation results after removing a single SNP are relatively stable and exhibit minor changes after the exclusion of a single SNP. Moreover, the causal estimates derived from the IVW method, after the removal of each SNP, do not demonstrate statistical significance, further supporting our previous conclusion. In summary, this Mendelian randomisation study provides support for the absence of a substantial causal link between IBD and T2DM.

**FIGURE 6:**
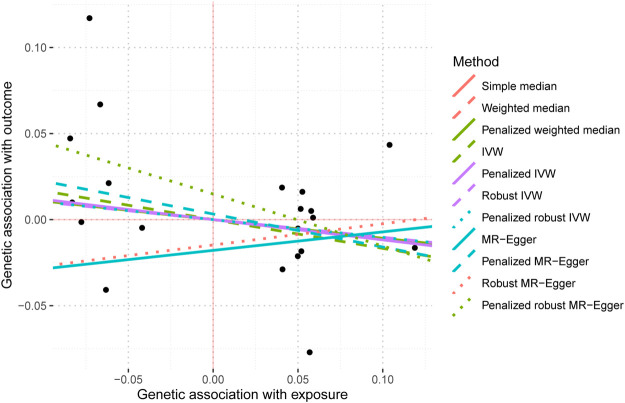
MR scatter plot diagram of the causal effect of two samples of T2DM on IBD.

The screening criteria for IVs were kept unchanged as described in the Methods section, resulting in 20 IVs (Supplementary Table S2). The outcomes acquired by the use of identical 11 MR techniques are presented in Table 4 and Figure 6. The lack of statistical significance and inconsistent positive and negative values of the predicted coefficients produced by all MR techniques can be observed in Table 4 and Figure 6. The aggregation of findings from many methodologies reveals that using MR analysis alone does not provide sufficient evidence to establish a causal relationship between T2DM and IBD.

**TABLE 4:** Estimation of causal effect of T2DM on IBD with different MR methods.

Method	Estimate	Std error	OR	95% CI		*p*-value
Simple median	−0.112	0.076	0.894	0.770	1.039	0.143
Weighted median	−0.104	0.077	0.901	0.774	1.048	0.178
Penalized weighted median	−0.108	0.077	0.897	0.771	1.044	0.161
IVW	−0.167	0.134	0.846	0.651	1.100	0.212
Penalized IVW	−0.118	0.062	0.889	0.787	1.004	0.058
Robust IVW	−0.103	0.156	0.902	0.665	1.224	0.509
Penalized robust IVW	−0.103	0.095	0.902	0.749	1.086	0.276
MR-Egger	0.108	0.507	1.113	0.412	3.007	0.832
Penalized MR-Egger	−0.192	0.232	0.826	0.524	1.300	0.409
Robust MR-Egger	0.123	0.482	1.131	0.440	2.909	0.798
Penalized robust MR-Egger	−0.302	0.277	0.739	0.429	1.273	0.276

* The ORs, express effects of liability to T2DM, on IBD, risk. OR, odds ratio; CI, confidence interval; MR, mendelian randomization.

**FIGURE 7:**
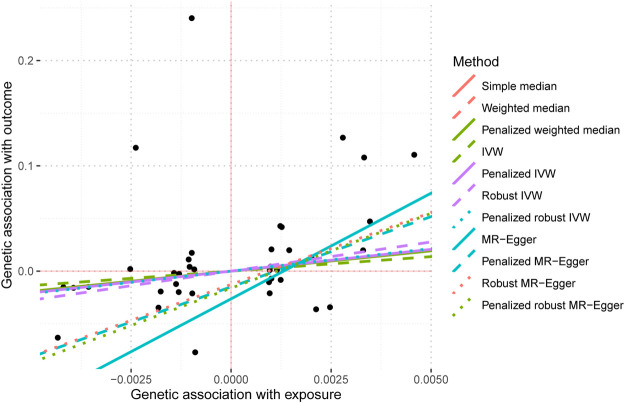
MR Scatter plot diagram of two samples of the causal Effect of IBS on IBD.

The present study employed irritable bowel syndrome (IBS) as the exposure variable while considering IBD and T2DM as the outcome variables to conduct a two-sample analysis. The Supplementary Material containing the MR imaging data for IBS in IBD can be accessed in Supplementary Table S3. The causative estimates derived from these data are presented in Table 5 and Figure 7. The acquired estimated values using several enhanced MR Egger techniques demonstrate statistical significance, suggesting a causal relationship between IBS and IBD.

**TABLE 5:** Estimation of causal effect of IBS on IBD with different MR methods.

Method	Estimate	Std error	OR	95% CI		*p*-value
Simple median	4.023	2.693	55.880	0.285	1.095E+04	0.135
Weighted median	4.010	2.677	55.160	0.290	1.048E+04	0.134
Penalized weighted median	3.851	2.648	47.020	0.262	8.444E+03	0.146
IVW	2.750	6.916	15.638	0.000	1.204E+07	0.691
Penalized IVW	4.133	2.025	62.377	1.179	3.301E+03	0.041
Robust IVW	5.537	2.888	253.827	0.884	7.286E+04	0.055
Penalized robust IVW	4.277	2.188	72.045	0.989	5.247E+03	0.051
MR-Egger	20.071	16.965	5.207E+08	0.000	1.438E+23	0.237
Penalized MR-Egger	13.337	4.543	6.196E+05	84.123	4.564E+09	0.003
Robust MR-Egger	13.642	6.029	8.408E+05	6.201	1.140E+11	0.024
Penalized robust MR-Egger	14.198	5.371	1.466E+06	39.318	5.465E+10	0.008

* The ORs, express effects of liability to IBS, on IBD, risk. OR, odds ratio; CI, confidence interval; MR, mendelian randomization.

**FIGURE 8:**
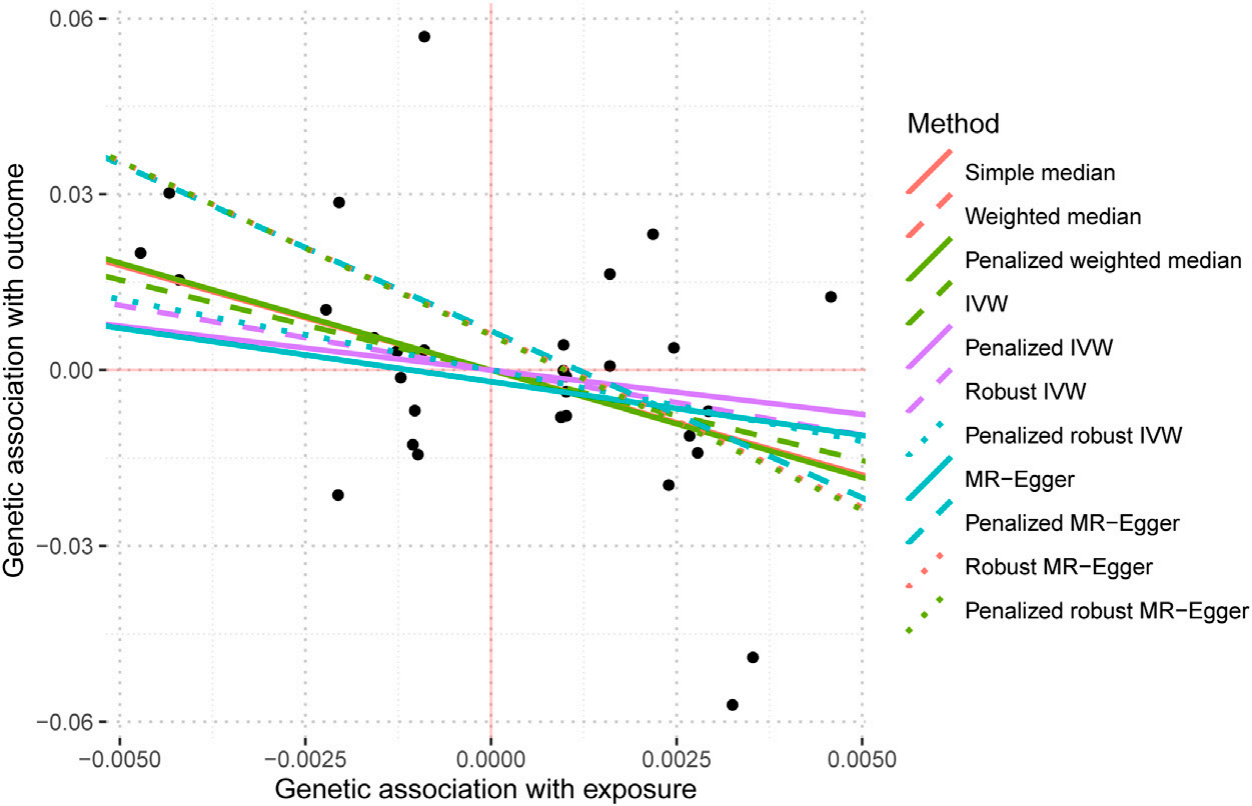
MR Scatter plot Diagram of Two Samples of the Causal Effect of IBS on T2DM.

The data used for MR imaging of IBS in T2DM can be found in Supplementary Table S4. The resulting causal estimates derived from this data are presented in Table 6 and Figure 8. It is evident that despite the negative estimated values derived from different methodologies, they lack statistical significance. Consequently, asserting that IBS may have a substantial causal impact on T2DM is unacceptable.

**TABLE 6:** Estimation of causal effect of IBS on T2DM with different MR methods.

Method	Estimate	Std error	OR	95% CI		*p*-value
Simple median	−3.573	1.988	0.028	0.001	1.380	0.072
Weighted median	−3.601	1.992	0.027	0.001	1.354	0.071
Penalized weighted median	−3.657	2.011	0.026	0.001	1.330	0.069
IVW	−3.082	2.205	0.046	0.001	3.458	0.162
Penalized IVW	−1.508	1.386	0.221	0.015	3.349	0.277
Robust IVW	−2.213	1.891	0.109	0.003	4.455	0.242
Penalized robust IVW	−2.420	2.131	0.089	0.001	5.791	0.256
MR-Egger	−1.826	5.229	0.161	0.000	4.54E+3	0.727
Penalized MR-Egger	−0.002	0.007	0.998	0.984	1.013	0.790
Robust MR-Egger	−5.690	3.239	0.003	0.000	1.932	0.079
Penalized robust MR-Egger	0.007	0.005	1.007	0.998	1.016	0.676

* The ORs, express effects of liability to IBS, on T2DM, risk. OR, odds ratio; CI, confidence interval; MR, mendelian randomization.

**FIGURE 9:**
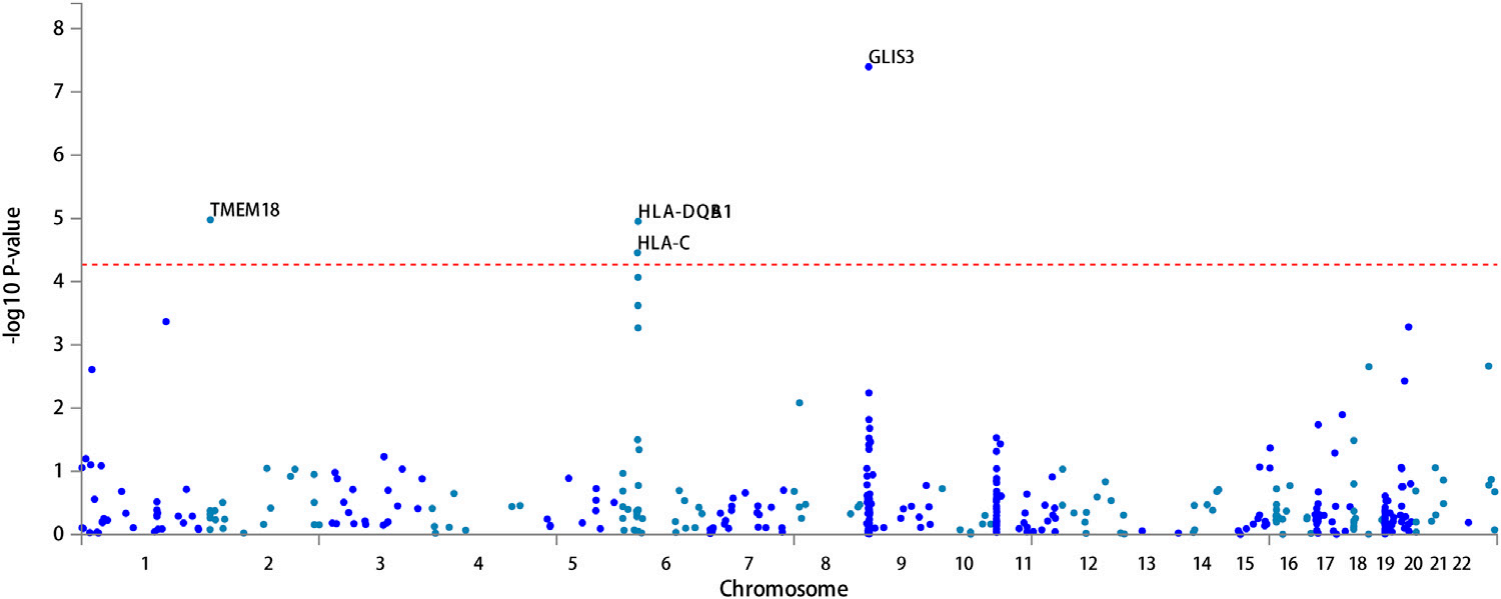
The GWAS Manhattan map of gene based for T2DM.

**FIGURE 10:**
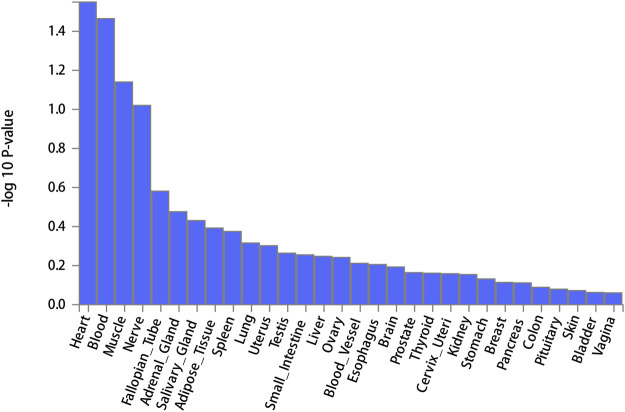
Expression of MAGMA gene in 30 general tissues.

**FIGURE 11:**
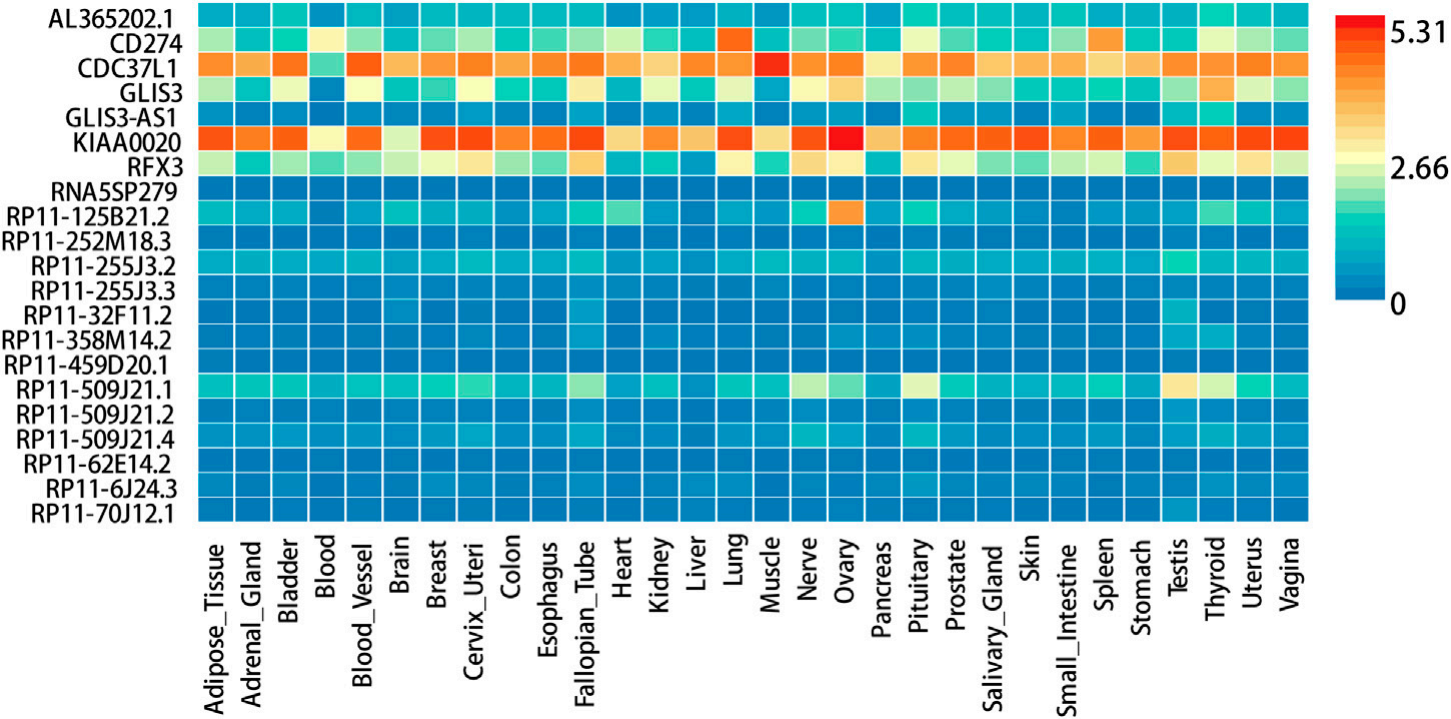
Expression heatmap of 22 fine mapped genes in 30 general tissues.

**FIGURE 12:**
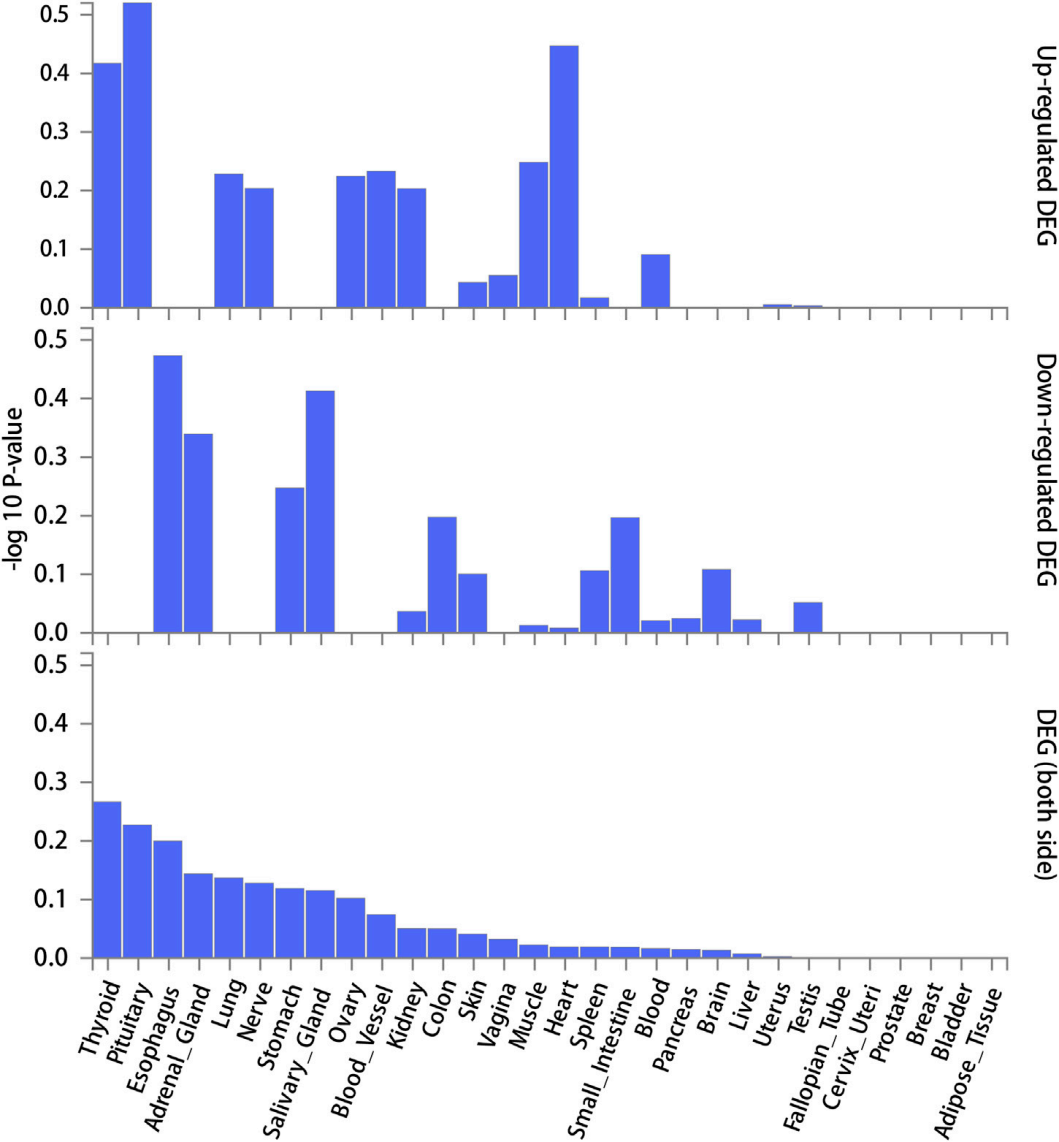
Enrichment testing of differentially expressed genes in 30 general tissues.

FUMA, a web-based bioinformatics tool that uses a combination of positional, eQTL and chromatin interaction mapping to prioritize likely causal variants and genes. The approach involved utilizing the acquired GWAS summary data for T2DM to conduct fine positional and functional analysis using the FUMA of the GWAS tool. The GWAS Manhattan map, which is based on genes, revealed the identification of four highly significant genes related to T2DM (Figure 9, Supplementary Table S4). FUMA precisely mapped 22 protein-coding genes utilizing significant SNPs acquired from GWAS. Table 7 displays the top 10 most significant gene sets enriched by the localized genes using MAGMA’s gene set enrichment analysis. The utilization of MAGMA to enhance the inclusion of genes into various tissues, predicated on their respective expression activity, is demonstrated in Figure 10. Several genes associated with T2DM do not exhibit notable tissue specificity. To further investigate the tissue-specific expression patterns of the genes linked with T2DM, we generated a heat map depicting their expression levels across various tissues (Figure 11). Additionally, we performed an enrichment analysis to assess the differential expression of these genes in distinct tissues (Figure 12). In line with the findings of MAGMA analysis, the genes associated with T2DM identified by fine mapping did not exhibit statistically significant variations in tissue-specific expression.

**TABLE 7:** MAGMA gene set enrichment analysis of the 10 most significant gene set information.

Gene set	N genes	Beta	Beta STD	Se	P	P_bon_
GOCC_ER_TO_GOLGI_TRANSPORT_VESICLE_MEMBRANE	9	2.2238	0.21887	0.43357	2.3546e-07	0.00174570044
GOCC_COPII_COATED_ER_TO_GOLGI_TRANSPORT_VESICLE	10	2.1426	0.22216	0.43095	5.0855e-07	0.00376988115
GOCC_COATED_VESICLE_MEMBRANE	19	1.6547	0.23533	0.3456	1.2202e-06	0.0090441224
WP_ALLOGRAFT_REJECTION	8	2.1954	0.20383	0.491	5.1758e-06	0.0383578538
GOCC_TRANSPORT_VESICLE_MEMBRANE	15	1.7589	0.22276	0.39617	5.9528e-06	0.044110248
GOMF_ANTIGEN_BINDING	10	2.1159	0.2194	0.4782	6.3574e-06	0.0471019766
GAURNIER_PSMD4_TARGETS	8	2.1457	0.19922	0.49165	8.2807e-06	0.0613434256
GOCC_LUMENAL_SIDE_OF_ENDOPLASMIC_RETICULUM_MEMBRANE	7	2.1309	0.18516	0.49267	9.8151e-06	0.0727004457
GOBP_ANTIGEN_PROCESSING_AND_PRESENTATION_OF_PEPTIDE_ANTIGEN	12	2.0629	0.23406	0.47816	1.0287e-05	0.076185522
GOCC_MHC_PROTEIN_COMPLEX	7	2.1245	0.18461	0.49275	1.0408e-05	0.07707124

This research utilizes publicly available GWAS summary information to examine the causal association between IBD, IBS, and T2DM using an MR method. Mendel’s randomized analysis revealed that the available data did not provide substantial support for a significant causal relationship between IBD and T2DM. Furthermore, the analysis did not find sufficient evidence to suggest a causal relationship between T2DM and IBD.

## Discussion

This was the first study to investigate the bidirectional causal association between IBD and T2DM using a comprehensive bidirectional two-sample MR analysis method. The results of the two-sample MR analysis did not provide any evidence to substantiate the association between genetically predicted IBD and T2DM in individuals of European ancestry. The findings from the reverse MR investigation also indicated a lack of evidence supporting a relationship between genetic susceptibility to T2DM and IBD. It is implying that the reported epidemiological associations of T2DM and IBD could be the result of unmeasured confounding factors or shared genetic architecture. To differentiate between an actual negative outcome and a potential lack of validity in the MR investigations, many sensitivity analyses were conducted to verify the fulfillment of the 3 MR assumptions. Given the consistency of our MR fndings across these diferent methods, we are confdent about the validity of our MR analyses to exclude moderate to large causal efects of the exposures on the outcomes. These analyses effectively rule out the presence of substantial causal effects of the exposures on the outcomes under investigation. Previous epidemiological studies that noted a link between IBD and T2DM have been undertaken (Kim et al., 2015; Abrahami et al., 2018; Villumsen et al., 2022). The results of the current study oppose an observational study by Abdo Jurjus et al., 2015 supporting an association between liability to IBD and increased risk of T2DM (Jurjus et al., 2015). Benchimol EI et al., 2015 conducted a thorough investigation to examine the genetic link between IBD and T2DM. The findings of that study revealed a favorable genetic correlation between these two conditions. The pathogenesis of IBD is complex and involves several factors. It is characterized by chronic and extensive inflammation in the gastrointestinal tract and an imbalance in the gut microbiota. This dysbiosis leads to the upregulation of various pro-inflammatory mediators and biomolecules, ultimately contributing to the development of T2DM (Benchimol et al., 2015). One plausible explanation for the observed correlations between IBD and T2DM, as indicated by our MR results, is the presence of pleiotropy in the lack of a causal relationship. In this study, we employed IBS as the exposure variable, while considering IBD and T2DM as the outcome variables to conduct a two-sample analysis. The results obtained using several improved MR Egger methodologies provide statistical significance (1.08; 95% CI, 1.03–1.12), suggesting a causative relationship between IBS and IBD. In a recent study, (Chen et al., 2023), showed an association between T2DM and IBS (1.08; 95% CI, 1.03–1.12). However, no substantial evidence supports a causal relationship between IBS and T2DM in our study. The differences in study results may be related to the sources of Biobank data.

In summary, the findings of the present investigation did not establish a direct relationship between genetically predicted IBD and T2DM. Similarly, there was no evidence of a causative association between genetically predicted T2DM and IBD. A potential association between IBD and T2DM may exist, wherein the gut microbiota might serve as a connecting factor. In a comparative study, untargeted metabolomics and shotgun metagenomic profiling were conducted on cross-sectional stool samples from two cohorts: a discovery cohort of 155 patients and a validation cohort of 65 patients. The patients in these cohorts were diagnosed with either Crohn’s disease (CD), ulcerative colitis (UC), or non-IBD controls. The study revealed enrichments of sphingolipids and bile acids and depletions of triacylglycerol and tetrapyrrole in these patients (Franzosa et al., 2019). Patients diagnosed with IBD exhibit a decrease in bacterial diversity and abundance compared to persons without the condition. This decrease is accompanied by an increase in the presence of Firmicutes and Bacteroidetes, which is comparable to the microbial composition observed in certain cases of T2DM (Quaglio et al., 2022; Lim et al., 2023). Numerous investigations have elucidated the role of ceramides and other sphingolipids in impeding the insulin-signaling pathway in skeletal muscles and the liver, contributing to insulin resistance and T2DM (Roszczyc-Owsiejczuk and Zabielski, 2021). Furthermore, a recent study has indicated that an increase in bile acids may result in an imbalance in the gut microbiota in T2DM model rats (Tawulie et al., 2023). The findings of this study provide evidence in favor of the notion that alterations in the composition of the intestinal microbiota and its associated metabolic profile in individuals with IBD may contribute to an elevated susceptibility to developing T2DM.

There are various limitations inherent in the current investigation. The study conducted in this research is based on European population data, and it is important to note that the findings should not be extrapolated to other populations. Furthermore, a number of our MR analyses were hindered by insufficient statistical power to identify subtle effects. This may be attributed to either the restricted variability of the exposures described by the SNP instruments or the low sizes of the outcome GWAS samples. The exclusion of ambiguous or palindromic SNPs from our MR instruments may have had further implications for the statistical power of this MR research. The utilization of comprehensive summary data from large-scale GWAS pertaining to IBD and T2DM is expected to enhance the statistical power of future MR investigations in identifying potential connections. Additionally, it is imperative to do a comprehensive multivariable MR study that encompasses IBD, T2DM, and gut microbiota.

## Data Availability

The datasets presented in this study can be found in online repositories. The names of the repository/repositories and accession number(s) can be found below: https://www.ebi.ac.uk/gwas/downloads/summary-statistics.

## References

[B1] Abrahami D. Douros A. Yin H. Yu O. H. Y. Renoux C. Bitton A. (2018). Dipeptidyl peptidase-4 inhibitors and incidence of inflammatory bowel disease among patients with type 2 diabetes: population based cohort study. BMJ 360, k872. 10.1136/bmj.k872 29563098 PMC5861502

[B2] Al Bander Z. Nitert M. D. Mousa A. Naderpoor N. (2020). The gut microbiota and inflammation: an overview. Int. J. Environ. Res. Public Health 17 (20), 7618. 10.3390/ijerph17207618 33086688 PMC7589951

[B3] Alemayehu E. Fiseha T. Bambo G. M. Sahile Kebede S. Bisetegn H. Tilahun M. (2023). Prevalence of hyperuricemia among type 2 diabetes mellitus patients in Africa: a systematic review and meta-analysis. BMC Endocr. Disord. 23 (1), 153. 10.1186/s12902-023-01408-0 37464401 PMC10353109

[B4] Alipour P. Senkevich K. Ross J. P. Spiegelman D. Manousaki D. Dion P. A. (2022). Investigation of the causal relationship between ALS and autoimmune disorders: a Mendelian randomization study. BMC Med. 20 (1), 382. 10.1186/s12916-022-02578-9 36320012 PMC9628014

[B5] Allin K. H. Agrawal M. Iversen A. T. Antonsen J. Villumsen M. Jess T. (2022). The risk of type 2 diabetes in patients with inflammatory bowel disease after bowel resections: a nationwide cohort study. Gastro Hep Adv. 1 (5), 777–784. 10.1016/j.gastha.2022.06.007 36117549 PMC9481066

[B6] Almigbal T. H. Alzarah S. A. Aljanoubi F. A. Alhafez N. A. Aldawsari M. R. Alghadeer Z. Y. (2023). Clinical inertia in the management of type 2 diabetes mellitus: a systematic review. Med. Kaunas. 59 (1), 182. 10.3390/medicina59010182 PMC9866102 36676805

[B7] Arsh A. Afaq S. Carswell C. Bhatti M. M. Ullah I. Siddiqi N. (2023). Effectiveness of physical activity in managing co-morbid depression in adults with type 2 diabetes mellitus: a systematic review and meta-analysis. J. Affect Disord. 329, 448–459. 10.1016/j.jad.2023.02.122 36868385

[B8] Benchimol E. I. Manuel D. G. To T. Mack D. R. Nguyen G. C. Gommerman J. L. (2015). Asthma, type 1 and type 2 diabetes mellitus, and inflammatory bowel disease amongst South Asian immigrants to Canada and their children: a population-based cohort study. PLoS One 10 (4), e0123599. 10.1371/journal.pone.0123599 25849480 PMC4388348

[B9] Bowden J. Davey Smith G. Haycock P. C. Burgess S. (2016). Consistent estimation in mendelian randomization with some invalid instruments using a weighted median estimator. Genet. Epidemiol. 40 (4), 304–314. 10.1002/gepi.21965 27061298 PMC4849733

[B10] Bruner L. P. White A. M. Proksell S. (2023). Inflammatory bowel disease. Prim. Care 50 (3), 411–427. 10.1016/j.pop.2023.03.009 37516511

[B11] Caioni G. Viscido A. d'Angelo M. Panella G. Castelli V. Merola C. (2021). Inflammatory bowel disease: new insights into the interplay between environmental factors and PPARγ. Int. J. Mol. Sci. 22 (3), 985. 10.3390/ijms22030985 33498177 PMC7863964

[B12] Cao Z. Wu Y. Li Q. Li Y. Wu J. (2022). A causal relationship between childhood obesity and risk of osteoarthritis: results from a two-sample Mendelian randomization analysis. Ann. Med. 54 (1), 1636–1645. 10.1080/07853890.2022.2085883 35703935 PMC9225762

[B13] Chen J. Yuan S. Fu T. Ruan X. Qiao J. Wang X. (2023). Gastrointestinal consequences of type 2 diabetes mellitus and impaired glycemic homeostasis: a mendelian randomization study. Diabetes Care 46 (4), 828–835. 10.2337/dc22-1385 36800530 PMC10091506

[B14] Dai C. Huang Y. H. Jiang M. (2023). Combination therapy in inflammatory bowel disease: current evidence and perspectives. Int. Immunopharmacol. 114, 109545. 10.1016/j.intimp.2022.109545 36508920

[B15] Dai Y. Liu X. Zhu Y. Mao S. Yang J. Zhu L. (2022). Exploring potential causal genes for uterine leiomyomas: a summary data-based mendelian randomization and FUMA analysis. Front. Genet. 13, 890007. 10.3389/fgene.2022.890007 35903355 PMC9315954

[B16] Da Rio L. Spadaccini M. Parigi T. L. Gabbiadini R. Dal Buono A. Busacca A. (2023). Artificial intelligence and inflammatory bowel disease: where are we going? World J. Gastroenterol. 29 (3), 508–520. 10.3748/wjg.v29.i3.508 36688019 PMC9850939

[B17] Ding M. Zhang Z. Chen Z. Song J. Wang B. Jin F. (2023). Association between periodontitis and breast cancer: two-sample Mendelian randomization study. Clin. Oral Investig. 27 (6), 2843–2849. 10.1007/s00784-023-04874-x PMC10264523 36749410

[B18] Dönertaş H. M. Fabian D. K. Valenzuela M. F. Partridge L. Thornton J. M. (2021). Common genetic associations between age-related diseases. Nat. Aging 1 (4), 400–412. 10.1038/s43587-021-00051-5 33959723 PMC7610725

[B19] Flynn S. Eisenstein S. (2019). Inflammatory bowel disease presentation and diagnosis. Surg. Clin. North Am. 99 (6), 1051–1062. 10.1016/j.suc.2019.08.001 31676047

[B20] Franzosa E. A. Sirota-Madi A. Avila-Pacheco J. Fornelos N. Haiser H. J. Reinker S. (2019). Gut microbiome structure and metabolic activity in inflammatory bowel disease. Nat. Microbiol. 4 (2), 293–305. 10.1038/s41564-018-0306-4 30531976 PMC6342642

[B21] Gagnon E. Mitchell P. L. Manikpurage H. D. Abner E. Taba N. Esko T. (2023). Impact of the gut microbiota and associated metabolites on cardiometabolic traits, chronic diseases and human longevity: a Mendelian randomization study. J. Transl. Med. 21 (1), 60. 10.1186/s12967-022-03799-5 36717893 PMC9887809

[B22] Gao L. Di X. Gao L. Liu Z. Hu F. (2023). The Frailty Index and colon cancer: a 2-sample Mendelian-randomization study. J. Gastrointest. Oncol. 14 (2), 798–805. 10.21037/jgo-23-134 37201057 PMC10186545

[B23] Gill P. A. Inniss S. Kumagai T. Rahman F. Z. Smith A. M. (2022). The role of diet and gut microbiota in regulating gastrointestinal and inflammatory disease. Front. Immunol. 13, 866059. 10.3389/fimmu.2022.866059 35450067 PMC9016115

[B24] Gordon H. Biancone L. Fiorino G. Katsanos K. H. Kopylov U. Al Sulais E. (2023). ECCO guidelines on inflammatory bowel disease and malignancies. J. Crohns Colitis 17 (6), 827–854. 10.1093/ecco-jcc/jjac187 36528797

[B25] Hands J. M. Corr P. G. Frame L. A. (2023). Clarifying the heterogeneity in response to vitamin D in the development, prevention, and treatment of type 2 diabetes mellitus: a narrative review. Int. J. Environ. Res. Public Health 20 (12), 6187. 10.3390/ijerph20126187 37372772 PMC10298297

[B26] Huang D. Lin S. He J. Wang Q. Zhan Y. (2022). Association between COVID-19 and telomere length: a bidirectional Mendelian randomization study. J. Med. Virol. 94 (11), 5345–5353. 10.1002/jmv.28008 35854470 PMC9349767

[B27] Jurjus A. Eid A. Al Kattar S. Zeenny M. N. Gerges-Geagea A. Haydar H. (2015). Inflammatory bowel disease, colorectal cancer and type 2 diabetes mellitus: the links. BBA Clin. 5, 16–24. 10.1016/j.bbacli.2015.11.002 27051585 PMC4802401

[B28] Kang N. Chen G. Tu R. Liao W. Liu X. Dong X. (2022). Adverse associations of different obesity measures and the interactions with long-term exposure to air pollutants with prevalent type 2 diabetes mellitus: the Henan Rural Cohort study. Environ. Res. 207, 112640. 10.1016/j.envres.2021.112640 34990613

[B29] Kim S. C. Schneeweiss S. Glynn R. J. Doherty M. Goldfine A. B. Solomon D. H. (2015). Dipeptidyl peptidase-4 inhibitors in type 2 diabetes may reduce the risk of autoimmune diseases: a population-based cohort study. Ann. Rheum. Dis. 74, 1968–1975. 10.1136/annrheumdis-2014-205216 24919467 PMC4263684

[B30] Li B. Wang J. Raza S. H. A. Wang S. Liang C. Zhang W. (2023). MAPK family genes' influences on myogenesis in cattle: genome-wide analysis and identification. Res. Vet. Sci. 159, 198–212. 10.1016/j.rvsc.2023.04.024 37148739

[B31] Li P. Wang H. Guo L. Gou X. Chen G. Lin D. (2022). Association between gut microbiota and preeclampsia-eclampsia: a two-sample Mendelian randomization study. BMC Med. 20 (1), 443. 10.1186/s12916-022-02657-x 36380372 PMC9667679

[B32] Lim S. Sohn M. Florez J. C. Nauck M. A. Ahn J. (2023). Effects of initial combinations of gemigliptin plus metformin compared with glimepiride plus metformin on gut microbiota and glucose regulation in obese patients with type 2 diabetes: the INTESTINE study. Nutrients 15 (1), 248. 10.3390/nu15010248 36615904 PMC9824054

[B33] Liu J. Z. van Sommeren S. Huang H. Ng S. C. Alberts R. Takahashi A. (2015). Association analyses identify 38 susceptibility loci for inflammatory bowel disease and highlight shared genetic risk across populations. Nat. Genet. 47 (9), 979–986. 10.1038/ng.3359 26192919 PMC4881818

[B34] Luo J. Xu Z. Noordam R. van Heemst D. Li-Gao R. (2022). Depression and inflammatory bowel disease: a bidirectional two-sample mendelian randomization study. J. Crohns Colitis 16 (4), 633–642. 10.1093/ecco-jcc/jjab191 34739073

[B35] Mahajan A. Wessel J. Willems S. M. Zhao W. Robertson N. R. Chu A. Y. (2018). Refining the accuracy of validated target identification through coding variant fine-mapping in type 2 diabetes. Nat. Genet. 50 (4), 559–571. 10.1038/s41588-018-0084-1 29632382 PMC5898373

[B36] Noviello D. Mager R. Roda G. Borroni R. G. Fiorino G. Vetrano S. (2021). The IL23-IL17 immune Axis in the treatment of ulcerative colitis: successes, defeats, and ongoing challenges. Front. Immunol. 12, 611256. 10.3389/fimmu.2021.611256 34079536 PMC8165319

[B37] Parigi T. L. Iacucci M. Ghosh S. (2022). Blockade of IL-23: what is in the pipeline? J. Crohns Colitis 16 (2), ii64–ii72. 10.1093/ecco-jcc/jjab185 35553666 PMC9097679

[B38] Pirinen M. Donnelly P. Spencer C. (2013). Efficient computation with a linear mixed model on large-scale data sets with applications to genetic studies. Ann. Appl. Stat. 7, 369–390. 10.1214/12-aoas586

[B39] Quaglio A. E. V. Grillo T. G. De Oliveira E. C. S. Di Stasi L. C. Sassaki L. Y. (2022). Gut microbiota, inflammatory bowel disease and colorectal cancer. World J. Gastroenterol. 28 (30), 4053–4060. 10.3748/wjg.v28.i30.4053 36157114 PMC9403435

[B40] Roszczyc-Owsiejczuk K. Zabielski P. (2021). Sphingolipids as a culprit of mitochondrial dysfunction in insulin resistance and type 2 diabetes. Front. Endocrinol. (Lausanne) 12, 635175. 10.3389/fendo.2021.635175 33815291 PMC8013882

[B41] Saez A. Herrero-Fernandez B. Gomez-Bris R. Sánchez-Martinez H. Gonzalez-Granado J. M. (2023). Pathophysiology of inflammatory bowel disease: innate immune system. Int. J. Mol. Sci. 24 (2), 1526. 10.3390/ijms24021526 36675038 PMC9863490

[B42] Sun H. Saeedi P. Karuranga S. Pinkepank M. Ogurtsova K. Duncan B. B. (2022). IDF Diabetes Atlas: global, regional and country-level diabetes prevalence estimates for 2021 and projections for 2045. Diabetes Res. Clin. Pract. 183, 109119. 10.1016/j.diabres.2021.109119 34879977 PMC11057359

[B43] Taborda Restrepo P. A. Acosta-Reyes J. Estupiñan-Bohorquez A. Barrios-Mercado M. A. Correa Gonzalez N. F. Taborda Restrepo A. (2023). Comparative analysis of clinical practice guidelines for the pharmacological treatment of type 2 diabetes mellitus in Latin America. Curr. Diab Rep. 23 (6), 89–101. 10.1007/s11892-023-01504-4 37126189 PMC10160131

[B44] Tawulie D. Jin L. Shang X. Li Y. Sun L. Xie H. (2023). Jiang-Tang-San-Huang pill alleviates type 2 diabetes mellitus through modulating the gut microbiota and bile acids metabolism. Phytomedicine 113, 154733. 10.1016/j.phymed.2023.154733 36870307

[B45] Tseng C. H. (2021). Metformin use is associated with a lower risk of inflammatory bowel disease in patients with type 2 diabetes mellitus. J. Crohns Colitis 15 (1), 64–73. 10.1093/ecco-jcc/jjaa136 32604412

[B46] Tseng C. H. (2022). Pioglitazone has a null association with inflammatory bowel disease in patients with type 2 diabetes mellitus. Pharm. (Basel) 15 (12), 1538. 10.3390/ph15121538 PMC9785412 36558989

[B47] Tseng C. H. (2023). Rosiglitazone does not affect the risk of inflammatory bowel disease: a retrospective cohort study in Taiwanese type 2 diabetes patients. Pharm. (Basel) 16 (5), 679. 10.3390/ph16050679 PMC10223056 37242462

[B48] Verdugo-Meza A. Ye J. Dadlani H. Ghosh S. Gibson D. L. (2020). Connecting the dots between inflammatory bowel disease and metabolic syndrome: a focus on gut-derived metabolites. Nutrients 12 (5), 1434. 10.3390/nu12051434 32429195 PMC7285036

[B49] Villumsen M. Poulsen G. Andersen N. N. Andersson M. Jess T. Allin K. H. (2022). Anti-tumor necrosis factor treatment does not decrease the risk of type 2 diabetes in patients with inflammatory bowel disease. Clin. Gastroenterol. Hepatol. 21, 3182–3184.e3. 10.1016/j.cgh.2022.12.011 36566818

[B50] Xian W. Wu D. Liu B. Hong S. Huo Z. Xiao H. (2023). Graves disease and inflammatory bowel disease: a bidirectional mendelian randomization. J. Clin. Endocrinol. Metab. 108 (5), 1075–1083. 10.1210/clinem/dgac683 36459455 PMC10099169

[B51] Xiang K. Wang P. Xu Z. Hu Y. Q. He Y. S. Chen Y. (2021). Causal effects of gut microbiome on systemic lupus erythematosus: a two-sample mendelian randomization study. Front. Immunol. 12, 667097. 10.3389/fimmu.2021.667097 34557183 PMC8453215

[B52] Xu J. Zhang S. Tian Y. Si H. Zeng Y. Wu Y. (2022). Genetic causal association between iron status and osteoarthritis: a two-sample mendelian randomization. Nutrients 14 (18), 3683. 10.3390/nu14183683 36145059 PMC9501024

[B53] Xu S. Li X. Zhang S. Qi C. Zhang Z. Ma R. (2023). Oxidative stress gene expression, DNA methylation, and gut microbiota interaction trigger Crohn's disease: a multi-omics Mendelian randomization study. BMC Med. 21 (1), 179. 10.1186/s12916-023-02878-8 37170220 PMC10173549

[B54] Yang Y. Knol M. J. Wang R. Mishra A. Liu D. Luciano M. (2023). Epigenetic and integrative cross-omics analyses of cerebral white matter hyperintensities on MRI. Brain 146 (2), 492–506. 10.1093/brain/awac290 35943854 PMC9924914

[B55] Yin K. J. Huang J. X. Wang P. Yang X. K. Tao S. S. Li H. M. (2022). No genetic causal association between periodontitis and arthritis: a bidirectional two-sample mendelian randomization analysis. Front. Immunol. 13, 808832. 10.3389/fimmu.2022.808832 35154127 PMC8825874

[B56] Yuan S. Chen J. Ruan X. Sun Y. Zhang K. Wang X. (2023a). Smoking, alcohol consumption, and 24 gastrointestinal diseases: mendelian randomization analysis. Elife 12, e84051. 10.7554/eLife.84051 36727839 PMC10017103

[B57] Yuan S. Wang L. Zhang H. Xu F. Zhou X. Yu L. (2023b). Mendelian randomization and clinical trial evidence supports TYK2 inhibition as a therapeutic target for autoimmune diseases. EBioMedicine 89, 104488. 10.1016/j.ebiom.2023.104488 36842216 PMC9988426

[B58] Zhang Q. Zhang K. Zhu Y. Yuan G. Yang J. Zhang M. (2023). Exploring genes for immunoglobulin A nephropathy: a summary data-based mendelian randomization and FUMA analysis. BMC Med. Genomics 16 (1), 16. 10.1186/s12920-023-01436-8 36709307 PMC9884184

[B59] Zhao G. Lu H. Liu Y. Zhao Y. Zhu T. Garcia-Barrio M. T. (2021). Single-cell transcriptomics reveals endothelial plasticity during diabetic atherogenesis. Front. Cell Dev. Biol. 9, 689469. 10.3389/fcell.2021.689469 34095155 PMC8170046

[B60] Zhou Z. Sun B. Yu D. Zhu C. (2022). Gut microbiota: an important player in type 2 diabetes mellitus. Front. Cell Infect. Microbiol. 12, 834485. 10.3389/fcimb.2022.834485 35242721 PMC8886906

