# Supplementary material for: Investigation of the causal relationship between inflammatory bowel disease and type 2 diabetes mellitus: a Mendelian randomization study
Source: Front Genet. 2024 Feb 16;15:1325401. doi: 10.3389/fgene.2024.1325401 (PMC10904574; doi:10.3389/fgene.2024.1325401)
Supplement: Supplementary file 1 [file DataSheet1.docx]

**Supplementary Table 1** Information of instrumental variables (IVs) subjected to inflammatory bowel disease (IBD).T2DM on IBD.

| Rs number | Chromosome | location | Other allele | Effect allele | MAF | P-value |
| --- | --- | --- | --- | --- | --- | --- |
| rs340874 | 1 | 213985913 | T | C | 0.53 | 5.10E-03 |
| rs2943641 | 2 | 226229029 | T | C | 0.64 | 5.89E-04 |
| rs1260326 | 2 | 27508073 | T | C | 0.61 | 5.69E-04 |
| rs35720761 | 2 | 43292838 | C | T | 0.11 | 7.29E-03 |
| rs11708067 | 3 | 123346931 | A | G | 0.22 | 7.77E-04 |
| rs6795735 | 3 | 64719689 | C | T | 0.40 | 4.19E-03 |
| rs35658696 | 5 | 103003107 | A | G | 0.05 | 1.18E-04 |
| rs7756992 | 6 | 20679478 | A | G | 0.29 | 1.04E-03 |
| rs1077394 | 6 | 31642607 | C | T | 0.66 | 5.20E-04 |
| rs2191349 | 7 | 15024684 | G | T | 0.53 | 5.26E-04 |
| rs864745 | 7 | 28140937 | T | C | 0.49 | 6.32E-04 |
| rs4607517 | 7 | 44196069 | G | A | 0.16 | 5.78E-06 |
| rs13266634 | 8 | 117172544 | C | T | 0.32 | 8.31E-07 |
| rs10758593 | 9 | 4292083 | G | A | 0.41 | 4.09E-04 |
| rs60980157 | 9 | 136340958 | C | T | 0.25 | 6.66E-06 |
| rs5215 | 11 | 17387083 | C | T | 0.61 | 6.16E-05 |
| rs1727307 | 12 | 123091195 | A | G | 0.72 | 4.06E-04 |
| rs10146997 | 14 | 79478819 | A | G | 0.22 | 5.16E-04 |
| rs7202877 | 16 | 75213347 | T | G | 0.10 | 8.43E-05 |
| rs17782313 | 18 | 60183864 | T | C | 0.23 | 4.99E-04 |

* SNPs location information using the human reference genome GRCH38 version.

**Supplementary Table 2** Information of instrumental variables (IVs) subjected to IBS on IBD.

| Rs number | Chromosome | location | Other allele | Effect allele | MAF | P-value |
| --- | --- | --- | --- | --- | --- | --- |
| rs3813965 | 1 | 206634569 | G | A | 0.37 | 3.18E-04 |
| rs316852 | 1 | 242212017 | T | C | 0.53 | 3.08E-04 |
| rs2201841 | 1 | 67228519 | A | G | 0.32 | 3.31E-04 |
| rs72702268 | 1 | 155073926 | G | T | 0.22 | 1.05E-03 |
| rs3102463 | 1 | 244381156 | A | G | 0.59 | 3.23E-04 |
| rs1260326 | 2 | 27508073 | T | C | 0.61 | 3.15E-04 |
| rs17031887 | 2 | 60687594 | T | C | 0.21 | 3.77E-04 |
| rs11695623 | 2 | 218203392 | G | A | 0.91 | 5.39E-04 |
| rs4450763 | 3 | 27765361 | T | C | 0.46 | 3.08E-04 |
| rs7655073 | 4 | 26035003 | G | A | 0.02 | 1.28E-03 |
| rs1813006 | 4 | 102080492 | G | T | 0.06 | 6.75E-04 |
| rs77945719 | 4 | 121986662 | C | T | 0.02 | 1.13E-03 |
| rs2560665 | 5 | 71698087 | A | G | 0.59 | 3.13E-04 |
| rs153643 | 5 | 116504511 | C | T | 0.92 | 5.72E-04 |
| rs115366841 | 5 | 160471922 | G | A | 0.03 | 8.93E-04 |
| rs13188563 | 5 | 161567799 | G | T | 0.06 | 6.51E-04 |
| rs6932387 | 6 | 4515681 | G | A | 0.32 | 3.30E-04 |
| rs2247056 | 6 | 31297713 | T | C | 0.72 | 3.40E-04 |
| rs9451249 | 6 | 89584369 | T | C | 0.17 | 4.09E-04 |
| rs2069835 | 7 | 22728252 | T | C | 0.06 | 6.72E-04 |
| rs45571645 | 7 | 27095477 | G | T | 0.02 | 1.17E-03 |
| rs150607 | 7 | 28528401 | G | A | 0.58 | 3.12E-04 |
| rs76421761 | 7 | 75539171 | G | A | 0.02 | 1.09E-03 |
| rs10949621 | 7 | 157001019 | T | C | 0.11 | 4.93E-04 |
| rs4872007 | 8 | 22681366 | G | A | 0.67 | 3.28E-04 |
| rs998731 | 8 | 80183160 | C | T | 0.47 | 3.09E-04 |
| rs2513347 | 8 | 97068770 | G | A | 0.72 | 3.40E-04 |
| rs3933326 | 9 | 120871670 | A | G | 0.68 | 3.35E-04 |
| rs8463 | 10 | 6116449 | A | G | 0.15 | 4.27E-04 |
| rs76419861 | 10 | 6565060 | G | A | 0.04 | 8.05E-04 |
| rs4963127 | 11 | 540158 | A | G | 0.35 | 3.22E-04 |
| rs831627 | 11 | 33724501 | A | G | 0.96 | 7.93E-04 |
| rs17528736 | 12 | 55974734 | C | T | 0.04 | 8.01E-04 |
| rs11631508 | 15 | 87955235 | A | G | 0.31 | 3.33E-04 |
| rs144632542 | 17 | 42192498 | A | G | 0.01 | 1.53E-05 |
| rs11649883 | 17 | 74948877 | G | A | 0.49 | 3.14E-04 |
| rs79887530 | 18 | 12810898 | T | C | 0.02 | 1.00E-04 |
| rs35752582 | 18 | 45320617 | G | A | 0.29 | 3.39E-04 |
| rs11672396 | 19 | 16607902 | A | G | 0.11 | 4.82E-04 |

* SNPs location information using the human reference genome GRCH38 version.

**Supplementary Table 3** Information of instrumental variables (IVs) subjected to IBS on T2DM.

| Rs number | Chromosome | location | Other allele | Effect allele | MAF | P-value |
| --- | --- | --- | --- | --- | --- | --- |
|  |  |  |  |  |  |  |
| rs12407007 | 1 | 30417685 | C | T | 0.05 | 2.22E-05 |
| rs147797700 | 1 | 43431825 | G | A | 0.02 | 3.52E-06 |
| rs2201841 | 1 | 67228519 | A | G | 0.32 | 9.85E-04 |
| rs2233851 | 1 | 151343848 | C | T | 0.01 | 4.72E-04 |
| rs2782948 | 1 | 208217909 | C | T | 0.33 | 1.01E-12 |
| rs11119315 | 1 | 209611861 | G | A | 0.17 | 1.57E-09 |
| rs3122712 | 1 | 212625635 | A | G | 0.29 | 1.02E-08 |
| rs35737297 | 1 | 217431312 | T | C | 0.04 | 2.78E-08 |
| rs10802990 | 1 | 241744515 | T | C | 0.52 | 9.41E-04 |
| rs1260326 | 2 | 27508073 | T | C | 0.61 | 8.98E-04 |
| rs41286594 | 2 | 96799647 | C | T | 0.03 | 3.25E-04 |
| rs17727261 | 2 | 124524333 | C | T | 0.04 | 2.67E-06 |
| rs1920047 | 2 | 207150174 | T | C | 0.95 | 2.46E-05 |
| rs4345220 | 4 | 41637499 | A | G | 0.53 | 9.00E-04 |
| rs10517695 | 4 | 158915315 | C | T | 0.01 | 4.20E-04 |
| rs17853861 | 5 | 120686122 | C | A | 0.16 | 1.60E-03 |
| rs2247056 | 6 | 31297713 | T | C | 0.71 | 1.60E-03 |
| rs2327429 | 6 | 133888699 | T | C | 0.30 | 9.75E-04 |
| rs4895643 | 6 | 144450709 | A | G | 0.32 | 1.01E-03 |
| rs45571645 | 7 | 27095477 | G | T | 0.02 | 4.33E-04 |
| rs3750092 | 7 | 29884456 | A | G | 0.05 | 2.18E-04 |
| rs2230009 | 8 | 31064419 | G | A | 0.06 | 2.04E-04 |
| rs2274110 | 10 | 13197651 | A | G | 0.18 | 1.22E-05 |
| rs41307074 | 10 | 97164843 | G | A | 0.07 | 2.06E-04 |
| rs1139971 | 11 | 44618718 | A | G | 0.67 | 1.01E-04 |
| rs308757 | 11 | 88425697 | A | G | 0.57 | 1.05E-05 |
| rs117689747 | 11 | 124145351 | C | T | 0.03 | 2.92E-04 |
| rs1044474 | 15 | 40553753 | A | G | 0.45 | 9.77E-04 |
| rs4843467 | 16 | 86850214 | C | T | 0.19 | 1.26E-05 |
| rs78943308 | 17 | 36602169 | G | C | 0.04 | 2.39E-04 |
| rs144632542 | 17 | 42192498 | A | G | 0.01 | 4.57E-04 |
| rs7249094 | 19 | 8607115 | G | A | 0.38 | 9.50E-04 |

* SNPs location information using the human reference genome GRCH38 version.

**Supplementary Table 4** GWAS Manhattan map gene set enrichment analysis of four highly significant genes.

| Gene Set | N genes | Beta | Beta STD | SE | P | P_bon_ |
| --- | --- | --- | --- | --- | --- | --- |
| TMEM18 | 1 | 2.1467 | 0.18523 | 0.49628 | 6.2857e-06 | 0.04230421106 |
| HLA-DQA1 | 1 | 1.6638 | 0.19237 | 0.39564 | 8.1643e-05 | 0.00983867423 |
| HLA-C | 1 | 2.2265 | 0.22865 | 0.4329 | 4.5746e-07 | 0.0132535612 |
| GLIS3 | 1 | 2.0873 | 0.22457 | 0.4785 | 6.3581e-06 | 0.0452369014 |

* SNPs location information using the human reference genome GRCH38 version.
